# Catestatin in Acutely Decompensated Heart Failure Patients: Insights from the CATSTAT-HF Study

**DOI:** 10.3390/jcm8081132

**Published:** 2019-07-30

**Authors:** Josip A. Borovac, Duska Glavas, Zora Susilovic Grabovac, Daniela Supe Domic, Domenico D’Amario, Josko Bozic

**Affiliations:** 1 Department of Pathophysiology, University of Split School of Medicine, Soltanska 2, 21000 Split, Croatia; 2 Institute of Emergency Medicine of Split-Dalmatia County (ZHM SDZ), Spinciceva 1, 21000 Split, Croatia; 3 Clinic for Cardiovascular Diseases, University Hospital of Split, Spinciceva 1, 21000 Split, Croatia; 4 Department of Internal Medicine, University of Split School of Medicine, Soltanska 2, 21000 Split, Croatia; 5 Department of Medical Laboratory Diagnostics, University Hospital of Split, Spinciceva 1, 21000 Split, Croatia; 6 Department of Health Studies, University of Split, Rudjera Boskovica 35, P.P. 464, 21000 Split, Croatia; 7 Department of Cardiovascular and Thoracic Sciences, IRCCS Fondazione Policlinico A. Gemelli, Università Cattolica Sacro Cuore, Largo Francesco Vito 1, 00168 Rome, Italy

**Keywords:** acute myocardial infarction, biomarkers, catestatin, coronary artery disease, heart failure, heart failure decompensation, left ventricular ejection fraction, troponin, NT-proBNP, NYHA functional class

## Abstract

The role of catestatin (CST) in acutely decompensated heart failure (ADHF) and myocardial infarction (MI) is poorly elucidated. Due to the implicated role of CST in the regulation of neurohumoral activity, the goals of the study were to determine CST serum levels among ninety consecutively enrolled ADHF patients, with respect to the MI history and left ventricular ejection fraction (LVEF) and to examine its association with clinical, echocardiographic, and laboratory parameters. CST levels were higher among ADHF patients with MI history, compared to those without (8.94 ± 6.39 vs. 4.90 ± 2.74 ng/mL, *p* = 0.001). CST serum levels did not differ among patients with reduced, midrange, and preserved LVEF (7.74 ± 5.64 vs. 5.75 ± 4.19 vs. 5.35 ± 2.77 ng/mL, *p* = 0.143, respectively). In the multivariable linear regression analysis, CST independently correlated with the NYHA class (β = 0.491, *p <* 0.001), waist-to-hip ratio (WHR) (β = −0.237, *p* = 0.026), HbA1c (β = −0.235, *p* = 0.027), LDL (β = −0.231, *p* = 0.029), non-HDL cholesterol (β = −0.237, *p* = 0.026), hs-cTnI (β = −0.221, *p* = 0.030), and the admission and resting heart rate (β = −0.201, *p* = 0.036 and β = −0.242, *p* = 0.030), and was in positive association with most echocardiographic parameters. In conclusion, CST levels were increased in ADHF patients with MI and were overall associated with a favorable cardiometabolic profile but at the same time reflected advanced symptomatic burden (CATSTAT-HF ClinicalTrials.gov number, NCT03389386).

## 1. Introduction

Acute decompensated heart failure (ADHF) is a complex clinical syndrome associated with high morbidity, mortality, and healthcare expenditures [1,2,3]. During the acute decompensation event, a cascade of multiple cellular pathways is activated and this activity can be quantified by measuring levels of circulating biomarkers [4]. Decades ago, Viquerat et al. showed that levels of endogenous norepinephrine and dopamine are elevated in patients with congestive heart failure (HF), compared to the healthy controls, thus, reflecting an increased sympathetic nervous system (SNS) activity [5]. Importantly, among patients with established HF, activation of the SNS, renin-angiotensin-aldosterone system (RAAS), and T-cell-mediated immune response is higher among those with ischemic etiology of the disease compared to non-ischemic idiopathic dilated cardiomyopathy [6]. Finally, patients with the ischemic HF also exhibit a higher resting muscle sympathetic nerve activity than patients with non-ischemic HF [7]. On the other hand, catestatin (CST) is a pleiotropic cardioprotective peptide that counterbalances the negative effects of SNS by promoting vasodilation [8] and by inhibiting catecholamine secretion [9,10]. Previous studies have demonstrated that high levels of CST might reflect increased sympathoadrenal activity [11,12,13], are associated with increased mortality in HF [14,15], and are a marker of poor ventricular remodeling after myocardial infarction (MI) [16]. Furthermore, CST levels were associated with disease severity in HF [17,18] and were similar between patients with preserved (HFpEF) and reduced (HFrEF) left ventricular ejection fraction (LVEF) phenotypes [17].

Due to the compensatory actions of CST with respect to adverse neurohumoral activation that is particularly pronounced in ischemic disease, we hypothesized that circulating CST levels would likely be higher in ADHF patients with a previous MI, compared to those without MI. Moreover, since CST reflects increased SNS activity, we further hypothesized that CST levels might be higher in ADHF patients with more reduced LVEF, thus, we expected to see a positive correlation of CST levels with the functional burden of the disease, as assessed by the New York Heart Association (NYHA) classification. Finally, since associations of CST with relevant biochemical and echocardiographic markers in ADHF population are poorly elucidated, our secondary objectives were to investigate the relationship of CST serum levels with echocardiographic parameters of the left ventricle/left atrium and laboratory biomarkers that are commonly used in the workup of ADHF patients. While this part of the study was exploratory in nature, we expected to generally observe beneficial associations of CST with these parameters, based on the corroborations from previous preclinical/mechanistic studies.

For reasons stated above, we sought to determine and compare the CST serum levels between patients, with and without, the previous history of MI and across the whole LVEF phenotype spectrum (HFrEF, HFmrEF, and HFpEF). Secondly, we aimed to investigate the relationship of CST serum levels with the NYHA functional class, echocardiographic indices of the left ventricle/left atrium, and select laboratory biomarkers.

## 2. Materials and Methods

### 2.1. Study Population

This was a non-randomized clinical cross-sectional study with a prospective follow-up planned in future analyses. Between January 2018 and February 2019, a total of 118 consecutive patients presenting with signs and symptoms of heart failure at the emergency department (ED) were considered eligible for the study inclusion and were hospitalized at the Department of Cardiology.

**Figure 1:**
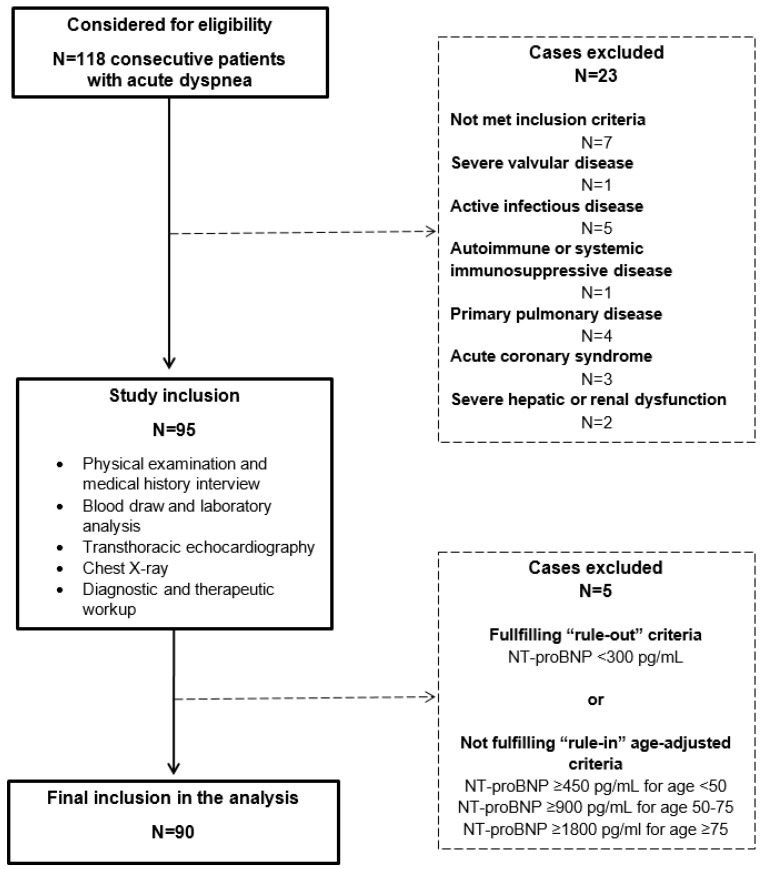
Flowchart of the CATSTAT-HF study.

In this study, we consecutively enrolled HF patients (of both sexes) with the New York Heart Association (NYHA) functional class II–IV and with a positive history of admission due to HF, who agreed to participate in the study and signed the informed written consent. The clinical and physical examination of the patients was undertaken according to the Framingham criteria for HF [19]. Diagnosis of the acute event due to heart failure was mandatorily confirmed as per the current European Society of Cardiology (ESC) guidelines, for the diagnosis and treatment of acute and chronic heart failure with the final diagnosis adjudicated and verified by the ESC-certified HF specialist who was on-site investigator for the study [20]. All patients received standard-of-care HF-directed treatment, according to their individual clinical status. In order to obtain a well-selected population with clear and undisputable cardiac etiology of dyspnea, a rigorous exclusion criteria were employed, which included adults <35 years and >90 years of age, patients with documented or newly-established severe valvular or pericardial disease, infiltrative or hypertrophic cardiomyopathy, cor pulmonale, primary pulmonary disease, diabetes mellitus type I, primary renal or hepatic disease, active malignant or infectious disease, systemic autoimmune disease, hemorrhagic diathesis or significant coagulopathy, systemic immunological, or an immunosuppressive disorder, or a positive recent history of immunosuppressive/cancer chemotherapeutic drug use, positive history of acute coronary syndrome or stroke within 3 months prior to study enrolment, positive history of excessive alcohol, drug, narcotics or sedative consumption, and significantly debilitating psychiatric or neurological condition (Figure 1). Additionally, patients not included in the analysis were those that, at the ED or in-ward admission, had NT-proBNP levels <300 pg/mL as a ”rule-out“ strategy, while age-adjusted NT-proBNP cut-off values for the ”rule-in“ of heart failure diagnosis were applied as following—450 pg/mL for age <50 years, 900 pg/mL for 50–75 years, and 1,800 pg/mL for ≥75 years, based on the data from PRIDE [21] and International Collaborative of NT-proBNP [22] studies, later validated in the ICON-Reloaded study [23].

The study protocol was approved by the Ethics Committee of the University Hospital of Split (approval no. 2181-147-01/06/M.S.-17-2) and University of Split School of Medicine Ethics Committee. All medical procedures were undertaken as in accordance with the Declaration of Helsinki and its latest revision in 2013. A study was registered on 3 January 2018 at ClinicalTrials.gov registry before the enrolment of the first patient (Serum Catestatin Expression and Cardiometabolic Parameters in Patients with Congestive Heart Failure (CATSTAT-HF); ClinicalTrials.gov number NCT03389386).

### 2.2. Data Measurement

All patients were evaluated in the first 24 h of admission and this evaluation consisted of physical examination, medical history interview (via custom checklist questionnaire), antecubital venous blood sampling for laboratory analyses, peripheral arterial blood gas sampling, transthoracic echocardiography (TTE), chest X-ray imaging, and a standard 12-lead electrocardiography (ECG) recording. All patients were assessed for the medical history, demographic data (age and sex), body weight and height, body mass index (BMI), body surface area (BSA), waist-to-hip ratio (WHR) and pharmacotherapy use. Patients were specially assessed for the documented previous history of myocardial infarction (defined as non-ST segment myocardial infarction-NSTEMI or ST-elevation myocardial infarction-STEMI and verified by the medical records). For the body weight (kg) and height (cm) measurements we used a calibrated scale (Seca, Birmingham, UK) and the BMI was calculated by the body weight (kg) being divided by height-squared (m^2^). BSA was calculated using the Mosteller formula [24]. Waist circumference (cm) was measured while standing at the mid-point between the inferior tip of the ribcage and the superior aspect of the iliac crest, while hip circumference (cm) was measured at the point providing maximum circumference over the buttocks, using a tape measure. WHR was calculated by dividing the waist by the hip circumference. Heart rate (HR) at admission was recorded at the first medical contact in hospital by using a 12-lead ECG machine, while resting HR was recorded at the bedside when the patients were stable, by using the AliveCor® Kardia mobile device system (AliveCor Inc., Mountain View, CA, USA) attached to an Apple iPhone (Apple Inc., Cupertino, CA, USA).

### 2.3. Definitions

Patients with systolic blood pressure >140 mmHg or diastolic blood pressure >90 mmHg or those taking antihypertensive medications were considered to have arterial hypertension [25]. Dyslipidemia was defined as total cholesterol (TC) ≥5.0 mmol/L or low-density lipoprotein cholesterol (LDL-c) ≥3.0 mmol/L serum levels or current treatment with lipid-lowering agents. Non-high-density lipoprotein (HDL) cholesterol fraction was calculated by the formula = (total cholesterol − HDL cholesterol). Chronic kidney disease (CKD) was defined as an estimated glomerular filtration rate (eGFR) <60 mL/min/1.73 m^2^, calculated by the Chronic Kidney Disease Epidemiology Collaboration (CKD-EPI) formula [26]. Hyperuricemia was defined as uric acid plasma levels >337 µmol/L for women and >403 µmol/L for men, as per our institutional laboratory cutoffs. Diabetes mellitus was defined as plasma glycated hemoglobin (HbA1c) ≥6.5% or fasting plasma glucose ≥7.0 mmol/L, according to the American Diabetes Association (ADA) guidelines or current treatment with oral hypoglycemic and/or insulin agents [27]. Obesity was determined by the BMI ≥30 kg/m^2^ as defined by the World Health Organization (WHO) criteria. Patients with admission hemoglobin count <119 g/L for women and <138 g/L for men were considered to have anemia. Smoking was defined as current or former/past smoking. Left bundle branch block (LBBB) was defined based on the analysis of the available in-hospital ECG tracings, according to established recommendations [28]. For the evaluation of HF-related symptom burden, we used a New York Heart Association (NYHA) functional classification of heart failure, adjudicated by the same on-site principal investigator in collaboration with the HF-certified cardiology specialist.

### 2.4. Echocardiography

A transthoracic echocardiography (TTE) examination was performed on the same day as blood samples were collected. All measurements were taken while patients were at rest and in the left lateral decubitus position and by following recommendations for cardiac chamber quantification by echocardiography in adults endorsed by the American Society of Echocardiography (ASE) and the European Association of Cardiovascular Imaging (EACVI) [29]. A single on-site cardiology consultant with an expertise in ultrasonography performed all echocardiographic examinations. Measurements included quantification of left ventricle (LV) internal dimensions in diastole (LVEDd, mm), systole (LVESd, mm) in parasternal long-axis view, and M-mode tracing while LV volumes were measured in the apical four- and two-chamber views. LV ejection fraction (LVEF) was measured several times by the 2D biplane method, according to the modified Simpson’s rule and the average value was recorded [30]. Fractional shortening (FS, %) was derived from linear measurements obtained from 2D images. Posterior left ventricular wall thickness (LVPWd, mm) and interventricular septal thickness (IVSd, mm) were measured by the linear method. LV mass was calculated by the linear method and the Cube formula—LV Mass (g) = 0.8 × 1.04 × [(LVEDd + IVSd + LVPWd)^3^ − LVEDd^3^] + 0.6. Left ventricular mass index (LVMI) was calculated by dividing LV mass with body surface area (BSA) and expressed in g/m^2^. Left-ventricular remodeling was assessed by calculating relative wall thickness (RWT) that allowed for the further classification of the LV mass increase as either concentric hypertrophy (RWT > 0.42) or eccentric hypertrophy (RWT ≤ 0.42), while RWT was calculated by the formula RWT = (2 × LVPWd)/LVEDd. Left atrium (LA, mm) size quantification was performed in the parasternal long-axis view, perpendicular to the aortic root long axis at the end of the LV systole, while aortic root (Ao, mm) measurements were obtained in the parasternal long-axis view. LA/Ao represents the ratio of LA internal diameter and diameter of Ao. Echocardiographic parameters were used to aid in the diagnosis of HFpEF and to be considered for the HFpEF diagnosis, patients had to have symptoms and signs of HF, LVEF ≥50% and elevated natriuretic peptides further accompanied with relevant structural or functional heart alterations, as assessed by echocardiography. Structural abnormalities included left-ventricular hypertrophy, left atrial enlargement, or left ventricular mass index (LVMI) ≥115 g/m^2^ for men and ≥95 g/m^2^ for women. Significant functional alterations were considered if the ratio of the peak early mitral inflow velocity over the early diastolic mitral annular velocity (E/e’) ≥13 cm/s and mean early diastolic myocardial relaxation velocities (e’ septal and lateral wall) were <9 cm/s. Doppler estimation of pulmonary artery systolic pressure (PASP) >35 mmHg was also used to aid in the diagnosis. Where necessary, to unmask diastolic dysfunction and alter pseudonormal filling into impaired relaxation, a Valsalva maneuver was performed in patients.

All images were acquired using the Vivid 9 ultrasound system (GE Medical Systems, Milwaukee, WI, USA) and stored/analyzed on the Echo PAC workstation (EchoPac PC, version 112; GE Medical Systems, Milwaukee, WI, USA).

### 2.5. Laboratory Analyses

Blood was drawn after patients fasted from the antecubital vein by using a polyethylene butterfly needle and was stored into two vials, one with a clot activator (BD Vacutainer® CAT-Clot Activator Tube, 2.0 mL) and other containing anticoagulant K3 EDTA (BD Vacutainer® K3E 3.5 mg, 2.0 mL). These samples were then immediately transferred to the Department of Medical Laboratory Diagnostics, where they were further processed according to good laboratory practice. From one of the vials, blood was centrifuged (20 min at 2,000 rpm at 4 °C) and the obtained sera were divided into two aliquots and stored at a temperature of −80 °C, until analysis. All blood samples were analyzed in the same certified institutional biochemical laboratory, by using the standard laboratory procedures.

Catestatin levels in serum were determined by an enzyme-linked immunosorbent assay (ELISA), by using a commercially-available diagnostic kit (EK-053-27CE, EIA kit, Phoenix Pharmaceuticals Inc., Burlingame, CA, USA). According to the manufacturer’s instruction, the kit measurement range was 0–100 ng/mL. Reported sensitivity for catestatin was 0.05 ng/mL with a linear range of 0.05–0.92 ng/mL. Cross-reactivity with endogenous human catestatin peptide for this assay kit was 100% with the intra-assay and inter-assay coefficient of variability being <10% and <15%, respectively. High-sensitivity cardiac troponin I (hs-cTnI) concentrations were determined by using Abbot Diagnostics hs-cTnI assay (Abbott ARCHITECT ci16200 analyzer, Abbott, Chicago, IL, USA) with the upper limit of 99th percentile being 34.2 ng/L for men and 15.6 ng/L for women. The N-terminal (1–76) pro brain natriuretic peptide (NT-proBNP) concentrations were determined using Eclesys® Cobas e601 NT-proBNP assay via the electrochemiluminescense (ECLIA) method (Roche Diagnostics, Manheim, Germany). Hemoglobin A1c (HbA1c) levels were measured by using high-performance liquid chromatography (HPLC) (Tosoh G8, Tosoh Bioscience, Tokyo, Japan). Electrolyte levels were determined by the potentiometric method, while C-reactive protein (CRP) was determined by the immunoturbidimetric method on the Architect c16200 system (Abbott, Chicago, IL, USA). Complete blood count and differential blood count were determined by using standard flow-cytometry-based hematologic analyses (ADVIA 2120i, Siemens Healthcare, Erlangen, Germany). Fasting glucose, total cholesterol, high-density lipoprotein cholesterol (HDL-c), low-density lipoprotein cholesterol (LDL-c), triglycerides, blood urea nitrogen (BUN), creatinine, D-dimer, and uric acid concentrations were analyzed through the standard laboratory methods (ARCHITECT ci16200, Abbott, Chicago, IL, USA).

### 2.6. Statistical Analysis

Data were analyzed using SPSS Statistics for Windows® (version 25.0, IBM, Armonk, NY, USA) and Prism 6 for Windows® (version 6.01, GraphPad, La Jolla, CA, USA). Data were presented as mean ± standard deviation (SD) or median (interquartile range) based on the variable distribution normality or number (N) with percentage (%), within the particular category of interest. Normality of distribution for continuous variables was assessed with the Kolmogorov-Smirnov test. For differences between groups, an independent samples t-test was used for continuous variables with normal distribution, while the Mann–Whitney U test was used for continuous variables with non-normal distribution. The chi-squared (χ^2^) test was used to determine differences between groups in terms of categorical variables, while the Fisher Exact test was employed in cases where a group of interest had <5 cases. For comparisons of continuous variables among >2 groups of interest, a one-way ANOVA was used with a post-hoc Tukey test. Finally, a multivariable linear regression analysis with forward algorithm was applied to determine significant and independent correlates of the catestatin serum level, which was defined as a dependent continuous variable. This linear regression model was weighted by sex, previous history of MI, and covariate-adjusted for age, BMI, eGFR, arterial systolic blood pressure, and LVEF. To further minimize the chance of overfitting, separate multivariable linear regression analyses were undertaken for each variable of interest. In these analyses, univariate beta coefficient and its respective significance value was reported for every evaluated variable, while the unstandardized coefficient (B) with standard error (SE), standardized coefficient (β), t-statistics, and their associated *p*-value were reported only for those variables that retained significance in the multivariable model. A two-tailed *p*-value <0.05 was considered to be statistically significant.

## 3. Results

### 3.1. Patients’ Baseline Characteristics

Out of 118 consecutive patients that presented with acute dyspnea, a total of 90 patients were included in the final analysis, after applying the study exclusion criteria (Figure 1).

There was equal representation of sexes, with a slight predominance of women (N = 47, 52.2%) and the mean age of the population being 70.3 ± 10.2 years. The majority of patients were in the NYHA III functional class (62.2%), followed by NYHA IV (21.1%), while 15 (16.7%) were in the NYHA II class. Less than half of the patients (N = 40, 44.4%) had suffered MI in the past, 37 (41.1%) were diabetics, 34 (37.8%) were present or former smokers, while 84 (93.3%) had arterial hypertension. Atrial fibrillation was documented in 55.6% of patients, while more than half of the patients (51.1%) were in the CKD stage 3 or higher. Furthermore, 36 (40%) patients had at least one HF-related hospitalization during the previous year. Most of the patients had HFrEF phenotype (N = 39, 43.4%), followed by HFpEF (N = 31, 34.4%), while 20 (22.2%) had a midrange LVEF. Baseline characteristics of the patient cohort are available in Table 1.

### 3.2. ADHF Patients with a History of MI and Those Without

Patients with a history of MI (MI+) tended to be males and smokers; they had more advanced kidney diseases and functional disease burden (as assessed by the NYHA classification), more HF-related hospitalizations in the previous year and more pacemaker/ICD/resynchronization devices implanted, compared to patients without any history of MI (MI–) (Table 1).

Regarding pharmacotherapy use prior to hospital discharge, a higher use of aspirin and statins was recorded among MI+ patients, compared to those without a history of MI. Among all patients, the coverage of guideline-directed medical therapy was 77.8% for angiotensin-converting enzyme (ACE) inhibitors or angiotensin receptor blockers (ARBs), 90% for beta-blockers, 26.7% for sacubitril-valsartan, while mineralocorticoid receptor antagonists were used in 46.7% of all patients. Diuretics were used by the vast majority of patients (91.1%), whereas half of the patients were on anticoagulation medications (Table 1).

In terms of laboratory data, MI+ patients had higher serum creatinine (139.8 ± 70.4 vs. 99.9 ± 42.0 mmol/L, *p* = 0.001) and lower eGFR (49.2 ± 21.7 vs. 63.8 ± 25.7 mL/min/1.73 m^2^, *p* = 0.005) values, compared to the MI– group. Biomarker values of NT-proBNP and hs-cTnI were significantly higher among MI+ than MI– patients (5,227 (3,079–12,004) versus 2,286 (1,110–5,976) pg/mL, *p* = 0.008 and 35.8 (19.3–84.2) versus 16.0 (10.0–27.3) ng/L, *p* = 0.001, respectively). Furthermore, the average glycated hemoglobin value was significantly higher among MI+ compared to MI– patients (6.97 ± 1.50 vs. 6.33 ± 0.94 %, *p* = 0.017, respectively). Finally, MI+ patients had lower concentrations of total cholesterol (4.1 ± 1.3 vs. 4.7 ± 1.3 mmol/L, *p* = 0.030) and its HDL and LDL fractions [0.9 (0.8–1.1) versus 1.0 (0.9–1.2) mmol/L, *p* = 0.023 and 2.4 ±1.1 vs. 2.9 ± 1.1 mmol/L, *p* = 0.029, respectively) compared to the MI– patients (Table 2). 

No significant differences between two groups stratified by MI history were observed with regards to the echocardiographic parameters, while the mean LVEF in the total ADHF sample was 43.4 ± 16.4 percent (Table 3).

### 3.3. CST Serum Levels Stratified by the History of MI and Across Different LVEF Phenotypes

**Figure 2:**
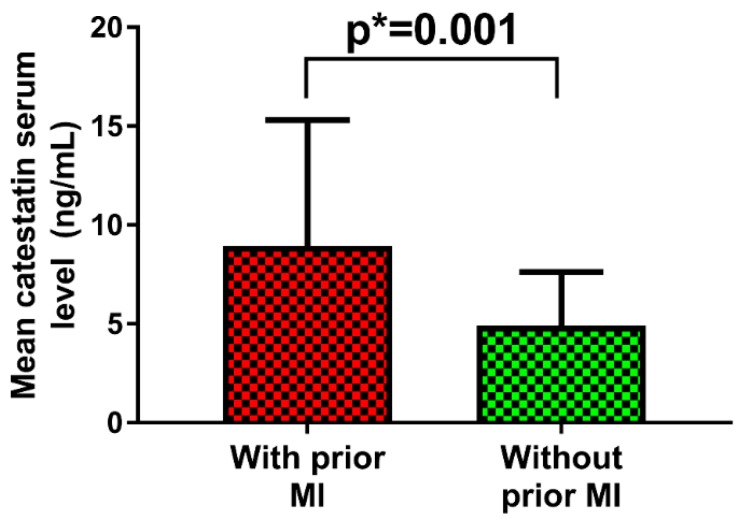
Catestatin (CST) serum levels in acutely decompensated heart failure patients stratified by the previous history of acute myocardial infarction.

When stratified by the history of myocardial infarction, MI+ patients had two-fold higher serum CST levels compared to MI– patients (8.94 ± 6.39 vs. 4.90 ± 2.74 ng/mL, *p* = 0.001) (Figure 2).

**Figure 3:**
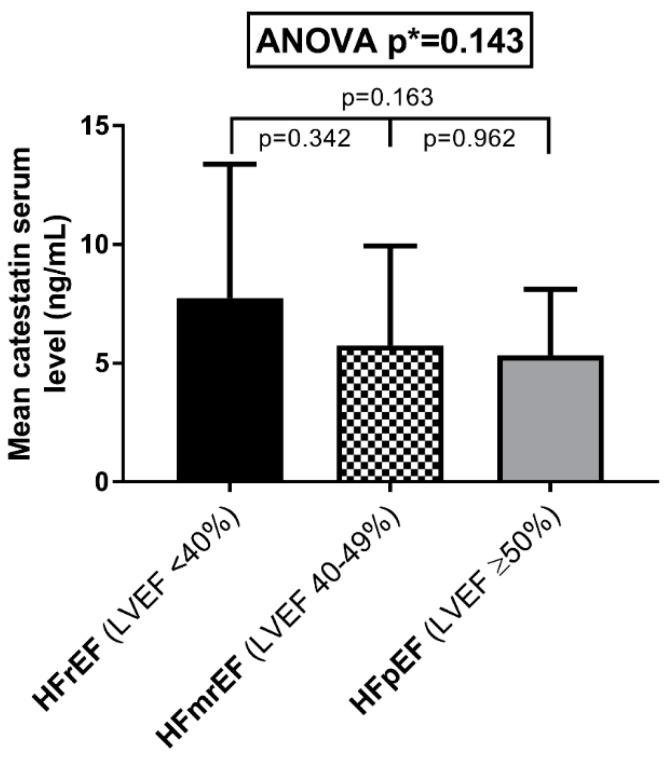
CST serum levels according to the left ventricular ejection fraction, stratified into three groups—heart failure with reduced ejection fraction (HFrEF), heart failure with midrange ejection fraction (HFmrEF), and heart failure with preserved ejection fraction (HFpEF).

CST serum levels did not significantly differ between the three LVEF phenotypes (*p* = 0.143). Patients in the HFrEF group exhibited the highest catestatin levels (7.74 ± 5.64 ng/mL), followed by the HFmrEF (5.75 ± 4.19 ng/mL) and HFpEF (5.35 ± 2.77 ng/mL) groups (Figure 3).

### 3.4. Associations of Serum CST Levels with Clinical and Laboratory Parameters

In multivariable linear regression analysis performed among the total patient sample, CST serum levels positively correlated with the NYHA functional class (β = 0.491, *p <* 0.001). Furthermore, CST levels were in inverse correlation with WHR (β = −0.237, *p* = 0.026), HbA1c (β = −0.235, *p* = 0.027), LDL (β = −0.231, *p* = 0.029), non-HDL cholesterol (β = −0.237, *p* = 0.026), and hs-cTnI (β = −0.221, *p* = 0.030) concentrations. Finally, heart rate, both at admission and measured at rest, negatively correlated with the CST serum level (β = −0.201, *p* = 0.036 and β = −0.242, *p* = 0.030, respectively) (Table 4). Each variable was tested in a multivariable linear regression model adjusted for covariates, with following univariate β estimates and p-values—age (β = −0.122, *p* = 0.320), BMI (β = −0.098, *p* = 0.801), eGFR (β = −0.109, *p* = 0.374), systolic blood pressure (β = 0.162, *p* = 0.412), LVEF (β = 0.311, *p* = 0.015), female sex (β = 0.249, *p* = 0.039), and previous history of MI (β = 0.378, *p <* 0.001).

### 3.5. Associations of Serum CST Levels with Echocardiographic Parameters

CST serum levels were in positive correlation with LVEF (β = 0.271, *p* = 0.022) and fractional shortening (β = 0.255, *p* = 0.029), while an inverse relationship was observed with respect to the left ventricular mass (β = −0.249, *p* = 0.031), left ventricular mass index (β = −0.237, *p* = 0.015), left ventricular end-diastolic (β = −0.341, *p* = 0.001) and end-systolic (β = −0.311, *p* = 0.005) diameters. Furthermore, left ventricular end-diastolic and end-systolic volumes, indexed to BSA, were in a negative correlation with the CST serum levels (β = −0.324, *p* = 0.002 and β = −0.328, *p* = 0.002, respectively). Finally, diameter of the left atrium inversely correlated with the CST serum levels (β = −0.255, *p* = 0.021) (Table 5).

**Figure 4:**
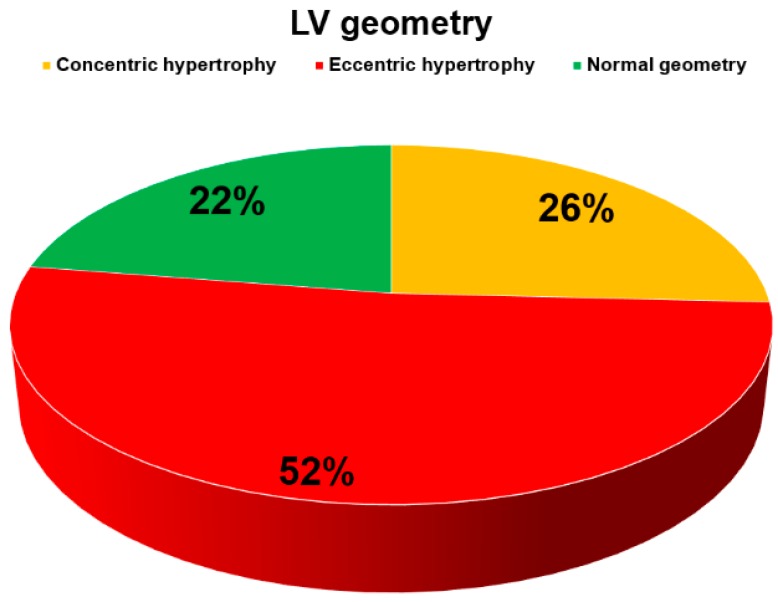
The distribution of left ventricular (LV) geometries as estimated by the relative wall thickness.

Finally, in terms of LV remodeling assessed by the value of relative wall thickness (RWT) derived from echocardiographic measurements, more than half of the patients (N = 47, 52.2%) had eccentric hypertrophy while one-quarter (N = 23, 25.8%) had concentric hypertrophy. Less than one-quarter of patients had normal left ventricular geometry (N = 20, 22.4%) (Figure 4).

## 4. Discussion

We report that, among ADHF patients, serum catestatin levels were higher by two-fold among individuals that previously had AMI, while no significant differences in catestatin serum levels were observed among the different HF phenotypes stratified by LVEF (HFrEF versus HFmrEF versus HFpEF). Furthermore, the catestatin serum levels positively correlated with the NYHA functional class, independent of other covariates, while they were in negative correlation with WHR, LDL, and non-HDL cholesterol, hs-cTnI, glycated hemoglobin, and heart rate (both at admission and at rest). Finally, the catestatin serum levels correlated favorably with most of the relevant structural and functional echocardiographic parameters of the left ventricle (LV), such as LVEF, fractional shortening, LV volumes and diameters, LV mass and mass index, as well as the size of the left atrium.

We hypothesized that, due to the elevated neurohumoral and SNS activation in patients with heart failure and ischemic heart disease [5,6,7,31], serum CST levels would likely be higher among patients that previously suffered AMI, compared to those that did not. Importantly, circulating CST levels closely paralleled norepinephrine levels in different myocardial ischemia states, among patients with CAD [32]. Furthermore, since catecholamines are co-stored and co-released with a group of acidic secretory proteins (such as ChgA), from the storage vesicles in adrenal chromaffin cells and adrenergic neurons [33,34], it is plausible that CST levels might closely relate to the catecholaminergic “milieu“ in the HF setting. As we hypothesized, our HF AMI+ cohort had more than two-fold higher levels of circulating catestatin, compared to the AMI– patients. This finding is comparable to another study of Liu et al. showing that plasma CST concentrations were significantly higher among HF patients with ischemic versus non-ischemic etiology of the disease, however, this finding was limited to patients in the NYHA III or IV class, and only HFpEF and HFrEF patients without a frank definition of ischemic disease [17].

Catestatin is a part of a complex neurohumoral feedback system and is most likely secreted peripherally as a counter-regulatory peptide attenuating excess catecholamine and SNS activity. Thus, higher levels of this peptide in peripheral blood might indirectly reflect increased neurohumoral burden and dysfunctional baroreflex control. However, precise pathophysiologic significance of the elevated CST levels in HF remains unknown [17,35]. Interestingly, this difference in catestatin serum levels between the AMI+ and AMI– cohort was persistent in our study, regardless of the baseline systolic blood pressure and guideline-directed pharmacotherapy targeting neurohumoral activation pathways, since both groups were similar in that regard. By measuring the circulating catestatin among similar HF patient groups (in terms of baseline characteristics and medication intake), it might be possible to quantify any “residual“ neurohumoral activity that could be additionally targeted by uptitration or introduction of additional neurohumoral disease-modifying drugs that might be used in HF. This could be relevant for hard clinical outcomes, since high circulating catestatin levels measured during index admission of CHF patients, independently predicted post-discharge all-cause death (HR 1.84, 95% CI 1.02–3.32) and cardiac death (OR 2.41 95% CI 1.26–4.62), while these effects were even stronger if the natriuretic peptide levels were high [14]. However, this finding must be cautiously interpreted, as it was based on a single-center study that enrolled 200 patients, thus, signaling that more studies with larger enrollment are required in order to further determine the prognostic value of CST as a biomarker implicated in HF.

Even more, CST measurement in combination with other disease biomarkers such as NT-proBNP might give additional prognostic information in the HF population [14]. Interestingly, in a study by Liu et al., plasma CST levels did not significantly decrease and remained high after treatment and alleviation of the HF-related symptoms, while BNP plasma levels were significantly reduced [17]. This finding might suggest that residual catecholaminergic and SNS activity is preserved in these patients, despite therapeutic intervention and subsequent short-term improvement of symptoms and reduction of the circulating natriuretic peptides. It might also suggest that CST is a chronic, rather than acute-response biomarker of neurohumoral activity in HF. Finally, multimarker strategies accumulating independent information from a battery of biomarkers in terms of risk stratification in ADHF and CHF are recommended nowadays, since each biomarker provides insight into different aspects of the HF pathophysiology [36,37].

In the present study, ADHF patients within the whole spectrum of LVEF were included and, to our knowledge, this is the first study that reported on CST levels in patients with “midrange“ LVEF. Although CST levels did not significantly differ between the three LVEF phenotypes, there is a clear trend towards HFrEF patients having higher CST levels, while statistical significance was not reached most likely due to the limited sample size. Additionally, circulating CST levels were highest in the NYHA IV, followed by NYHA III, and were lowest in the NYHA II subgroup. This finding was supported by a study of Liu et al. showing that plasma CST concentrations gradually increased among CHF patients from NYHA I to IV class and no difference in CST concentrations was observed between the HFrEF and HFpEF patients [17]. However, one study showed that the catestatin levels decreased gradually from stage A to C of HF, when using the American Heart Association (AHA) and American College of Cardiology (ACC) classification of HF [18]. In accordance with the previous study by Liu et al, our findings might suggest that CST is a marker of advanced HF and its circulating levels are dependent on a constellation of factors beyond the ejection fraction. Since the NYHA class is consistently associated with hospitalizations and mortality in HF [38,39,40], our finding is of further relevance because higher plasma CST levels measured at index hospitalization were independently associated to increased risk of all-cause and cardiac death among CHF patients, during the median follow-up of 52.5 months [14].

Importantly, our study showed that serum CST levels positively correlated with LVEF and fraction shortening. Higher catestatin levels were associated with smaller LV volumes and dimensions, as well as decreased LV mass and LA size. These are novel findings suggesting the cardioprotective association of catestatin with structural and functional properties of left ventricle and dimensions of the left atrium. Previous studies in which similar positive associations were observed were only reported in the rat model of AMI [41] and in patients with AMI [16], but not in the ADHF setting. One study conducted in the setting of AMI failed to demonstrate the relationship between catestatin and LVEF [42]. Furthermore, patients with essential hypertension and left ventricular hypertrophy, as determined by echocardiography, had a significantly lower catestatin-to-norepinephrine ratio [43], while mice with ablated ChgA gene showed increased LV mass and LV cavity dimensions. This suggests that deficiency of the prohormone ChgA and its downstream products such as catestatin are responsible for the loss of endogenous “brake“ on adrenergic activity and mediation of pathologic LV remodeling [44]. Unlike our findings, Liu et al. did not find LVEF as an independent correlate of plasma CST levels in their CHF cohort but rather reported the NYHA class, ischemic etiology of the cardiomyopathy, and eGFR as independent predictors of CST levels [17].

We further expanded on these findings since our multivariable regression analysis showed that independent negative predictors of CST serum levels were HbA1c, LDL and non-HDL cholesterol fractions, hs-cTnI, WHR, and HR (both at admission and at rest). These are novel findings in the context of HF and can be related to some reports on the role of CST in other diseases. Of note, catestatin suppressed hepatic glucose production and was associated with improved insulin sensitivity in mice with diet-induced obesity [45], while it also induced glucose uptake and GLUT4 trafficking in adult rat cardiomyocytes, thus, likely improving cardiac energetics [46]. One study performed among obese children and adolescents found that catestatin negatively correlated with the homeostatic model assessment of insulin resistance (HOMA-IR) and its levels were significantly reduced in obese subjects, compared to the control group [47]. CST also improved peripheral leptin sensitivity and promoted lipolysis and fatty acid oxidation in a preclinical model [48], thus, confirming its antiobesic effect [49]. These data altogether suggest that CST is associated with improved systemic glycemia and metabolic profile and these inferences are concordant to our findings in the ADHF population, since we observed negative associations of glycated hemoglobin and WHR with serum CST concentrations.

In terms of catestatin and lipid metabolism, no comparable data are available for the HF population. In a study among untreated hypertensive patients, catestatin levels correlated positively with HDL-cholesterol levels but no associations were observed with respect to the total cholesterol or other cholesterol fractions [50], while CST correlated negatively with HDL cholesterol among obstructive sleep apnea patients [11]. Similarly, catestatin significantly retarded aortic atherosclerotic lesions with declined lipoprotein-induced foam cell formation in a preclinical experiment [51], while its levels were inversely associated with the severity of atherosclerosis, among patients with CAD [52]. These data suggest that CST is associated with an improved lipid profile and likely exerts antiatherosclerotic effects, thus, partially explaining the negative associations of LDL and non-HDL cholesterol fractions with the CST levels, as observed in this study.

Relationship of CST with cardiac troponin, a hallmark biomarker of myocardial injury, has not been previously described, especially not in the HF setting. Our study revealed a weak but significant and independent inverse association of hs-cTnI concentrations with CST. Increased troponin levels in HF are common and can occur due to many mechanisms other than myocardial ischemia, such as elevated filling pressures, increased wall stress, tachycardias, arrhythmias, anemia, hypotension, and increased cardiomyocyte membrane permeability, causing a leak of cardiac troponin from the cytosolic pool into circulation [53]. Furthermore, pathologic processes in HF that occur on a cellular level, such as cardiomyocyte apoptosis, dysfunctional autophagy, and chronic breakdown of contractile apparatus, can result in a rise of circulating troponin levels, particularly those detected with high-sensitivity assays [53,54]. Since CST was associated with improved structural and functional echocardiographic parameters and lower left ventricular mass in our study, it is plausible that these effects might attenuate the extent of myocardial injury during the acute HF decompensation. However, it remains unknown whether this relationship is of chronic or acute nature and what are the dynamics of circulating troponin and CST during the natural course of HF.

Finally, CST serum levels were independently and inversely related to HR, both at admission and bedside rest. Previously, CST was characterized as an endocrine modulator of cardiac inotropism and lusitropism, by exhibiting cardiosuppressive effects on basal cardiac function and antagonistic action on beta-adrenergic positive inotropism and endothelin-1-mediated vasoconstriction, especially under stressed conditions [55,56,57]. Likewise, a study performed in mice showed that CST has a profound effect on autonomic function as its administration improved HR variability and decreased tachycardia by 10% in a ChgA knockout mice model, while wildtype mice had significantly lower HR at baseline compared to a ChgA knockout [58]. Our findings seem to support these preclinical observations, since patients with higher CST levels were more likely to have a generally lower HR.

There are several limitations to this study. This is a single-center clinical report with the cross-sectional design and no follow-up, therefore, we lack data on CST dynamics at various time-points and no causal inferences could be made due to the possibility of interference of non-measured confounders. Thus, enrolment of a larger number of patients might be required to generalize these results and to ascertain the potential relationship of CST with other relevant parameters of interest. Due to the lack of mechanistic insights, associations of CST with the measured parameters cannot be verified through a direct pathophysiological link, thereby, requiring further elucidation in future mechanistic preclinical and translational studies. Furthermore, direct measurements of circulating or urinary catecholamines or SNS activity parameters have not been performed in this study, therefore, we could not quantify catecholamine burden or sympathetic “excess“ in our ADHF cohort. Finally, we lack data on specific pulmonary function parameters and lifestyle-related parameters as these were not prespecified and measured in our study design.

## 5. Conclusions

Results of this study are novel for this population and suggest that the role of CST in HF is complex and multidimensional. On the one hand, higher CST levels are associated with beneficial metabolic and cardioprotective effects and, on the other, high CST levels reflect higher disease severity and most likely parallel increased neurohumoral activity that might translate to adverse outcomes during the natural course of disease. These findings could have clinical impact in the sense that CST could serve as a chronic marker of increased or residual sympathetic activation and could potentially be measured alongside established HF biomarkers, such as natriuretic peptides in the identification of HF patients with advanced disease burden and an increased risk of post-discharge mortality. Furthermore, future treatments developed to target neurohumoral pathways and to attenuate sympathetic nervous system activity in HF might translate to changes in circulating CST levels, thus, making them appropriate for the measurement of response to HF-directed therapy. Finally, further mechanistic studies and larger clinical studies with follow-up powered for relevant clinical endpoints are required to confirm the findings obtained in this study.

## Figures and Tables

**Table 1:** Baseline data and pharmacotherapy of the enrolled cohort stratified by the positive history of myocardial infarction.

VARIABLE	Total Cohort N = 90	ADHF without MI N = 50	ADHF with MI N = 40	*p*-Value *
**Demographics and clinical profile**				
Age, years	70.3 ± 10.2	69.8 ± 10.8	70.9 ± 9.6	0.610
Female sex	47 (52.2)	33 (66.0)	14 (35.0)	**0.003**
Body mass index, *kg/m^2^*	30.2 ± 4.2	30.8 ± 4.4	29.6 ± 3.9	0.182
Body surface area, *m^2^*	2.02 ± 0.18	2.02 ± 0.19	2.03 ± 0.17	0.792
Waist-to-hip ratio	0.98 ± 0.08	0.97 ± 0.09	0.99 ± 0.06	0.095
Systolic blood pressure, *mmHg*	137 ± 28	134 ± 23	140 ± 32	0.285
Diastolic blood pressure, *mmHg*	80 ± 13	79 ± 12	82 ± 14	0.279
Heart rate at admission, *beats/min*	95 ± 31	94 ± 28	96 ± 35	0.726
Heart rate at rest, *beats/min*	88 ± 26	90 ± 28	84 ± 22	0.300
HF-related hospitalization event in a previous year	36 (40.0)	15 (30.0)	21 (52.5)	**0.030**
Pacemaker/ICD/CRT device	13 (14.4)	3 (6.0)	10 (25.0)	**0.011**
Left bundle branch block	35 (38.9)	19 (38.0)	16 (40.0)	0.847
NYHA functional class	3.0 ± 0.62	2.9 ± 0.53	3.2 ± 0.69	**0.031**
CKD stage	2.6 ± 0.9	2.3 *± 1.0*	2.9 ± 0.8	**0.004**
LVEF ≤35%, *biplane Simpson*	37 (41.1)	16 (32.0)	20 (50.0)	0.083
SaO_2_ <90% at admission	34 (37.8)	18 (36.0)	10 (40.0)	0.777
**Comorbidities and concomitant clinical conditions**				
Diabetes mellitus	37 (41.1)	17 (34.0)	20 (50.0)	0.125
Anemia	36 (40.0)	12 (24.0)	14 (35.0)	0.253
Obesity	38 (42.2)	25 (55.6)	13 (35.1)	0.065
Hyperuricemia	75 (83.3)	40 (83.3)	35 (87.5)	0.193
Dyslipidemia	60 (66.6)	33 (66.6)	27 (67.5)	0.743
Chronic obstructive pulmonary disease	21 (23.3)	14 (28.0)	7 (17.5)	0.242
Chronic kidney disease	46 (51.1)	21 (42.0)	25 (62.5)	0.053
Arterial hypertension	84 (93.3)	45 (90.0)	39 (97.5)	0.156
Atrial fibrillation	50 (55.6)	28 (56.0)	22 (55.0)	0.924
Peripheral artery disease	19 (21.1)	9 (18.0)	10 (25.0)	0.419
Smoker, *present or former*	34 (37.8)	14 (28.0)	20 (50.0)	**0.032**
History of stroke or transient ischemic attack	7 (7.8)	4 (8.0)	3 (7.5)	0.930
**Pharmacotherapy**				
ACE inhibitor or ARB	70 (77.8)	41 (82.0)	29 (72.5)	0.245
Sacubitril-valsartan	24 (26.7)	11 (22.0)	13 (32.5)	0.154
β-blocker	81 (90.0)	43 (86.0)	38 (95.0)	0.235
Ca^2+^ channel blocker	13 (14.4)	9 (18.0)	4 (10.0)	0.305
MRA	42 (46.7)	24 (48.0)	18 (45.0)	0.953
Diuretics	82 (91.1)	45 (90.0)	37 (92.5)	0.274
Digoxin	18 (20.0)	9 (18.0)	9 (22.5)	0.554
Aspirin	35 (38.9)	13 (26.0)	22 (55.0)	**0.005**
Warfarin	23 (25.6)	14 (28.0)	9 (22.5)	0.617
NOAC	22 (24.4)	13 (26.0)	9 (22.5)	0.821
Statin	33 (36.7)	14 (28.0)	19 (47.5)	**0.035**

Values are mean ± SD or N (%); * an independent samples t-test or Chi-squared test or Fisher Exact test were used for comparisons between two groups of interest, as appropriate. Abbreviations: ACE—angiotensin-converting enzyme; ARB—angiotensin receptor blocker; CKD—chronic kidney disease; CRT—cardiac resynchronization therapy; HF—heart failure; ICD—implantable cardioverter defibrillator; LVEF—left ventricular ejection fraction; MI—myocardial infarction; MRA—mineralocorticoid receptor antagonist; NOAC—novel oral anticoagulant; NYHA—New York Heart Association; SaO_2_—peripheral arterial oxygen saturation. Bold: statistical significance <0.05.

**Table 2:** Laboratory data of the enrolled cohort stratified by the history of myocardial infarction.

Variable	Total Cohort N = 90	ADHF without Prior MI N = 50	ADHF with Prior MI N = 40	*p*-Value *
**Laboratory values**				
Sodium, mmol/L	138.9 ± 3.7	139.0 ± 4.1	138.7 ± 3.2	0.725
Potassium, mmol/L	4.2 ± 0.4	4.1 ± 0.4	4.2 ± 0.5	0.252
Creatinine, µmol/L	117.6 ± 59.5	99.9 ± 42.0	139.8 ± 70.4	**0.001**
BUN, mmol/L	4.9 ± 2.6	4.5 ± 2.5	5.4 ± 2.8	0.132
eGFR, mL/min/1.73 m^2^	57.3 ± 24.9	63.8 ± 25.7	49.2 ± 21.7	**0.005**
Uric acid, µmol/L	535 ± 165	511 ± 179	565 ± 143	0.130
Albumin, g/L	38.7 ± 4.1	38.6 ± 4.1	38.8 ± 4.2	0.849
Total proteins, g/L	68.2 ± 7.2	67.5 ± 7.8	69.0 ± 6.3	0.314
Hemoglobin, g/L	133.4 ± 19.2	134.1 ± 18.7	132.6 ± 19.9	0.706
WBC count, ×10^9^/L	8.08 ± 2.59	7.73 ± 2.49	8.51 ± 2.66	0.156
Erythrocytes, ×10^12^/L	4.48 ± 0.69	4.46 ± 0.69	4.51 ± 0.71	0.775
Thrombocytes, ×10^9^/L	214 ± 60	217 ± 61	209 ± 58	0.522
Lymphocytes, ×10^9^/L	1.50 ± 1.35	1.49 ± 0.70	1.50 ± 0.68	0.970
Neutrophils, ×10^9^/L	5.64 ± 2.18	5.41 ± 2.11	5.92 ± 2.24	0.278
Fasting glucose, mmol/L	8.2 ± 3.0	7.7 ± 2.6	8.8 ± 3.4	0.091
HbA1c, %	6.61 ± 1.26	6.33 ± 0.94	6.97 ± 1.50	**0.017**
NT-proBNP, pg/mL	3586 (1361–7787)	2286 (1110–5976)	5277 (3079–12004)	**0.008**
hs-cTnI, ng/L	22.9 (11.6–49.0)	16.0 (10.0–27.3)	35.8 (19.3–84.2)	**0.001**
CRP, mg/L	8.4 (4.9–20.5)	7.9 (4.7–17.7)	11.5 (6.6–30.6)	0.110
D-dimer, mg/L	1.62 ± 1.35	1.55 ± 1.33	1.72 ± 1.40	0.718
Triglycerides, mmol/L	1.56 ± 0.64	1.51 ± 0.64	1.61 ± 0.65	0.471
Total cholesterol, mmol/L	4.4 ± 1.3	4.7 ± 1.3	4.1 ± 1.3	**0.030**
HDL cholesterol, mmol/L	1.0 (0.8–1.2)	1.0 (0.9–1.2)	0.9 (0.8–1.1)	**0.023**
LDL cholesterol, mmol/L	2.7 ± 1.1	2.9 ± 1.1	2.4 ± 1.1	**0.029**

Values are mean ± SD or median (interquartile range); * Based on the variable distribution, an independent samples *t*-test or Mann–Whitney U test were used for the group comparisons, as appropriate. Abbreviations: BUN–blood urea nitrogen; CRP–C-reactive protein; eGFR–estimated glomerular filtration rate by chronic kidney disease epidemiology collaboration (CKD–EPI) formula; HDL–high-density lipoprotein; HbA1c–glycated hemoglobin; hs-cTnI–high sensitivity cardiac troponin I; LDL–low-density lipoprotein; NTproBNP—N-terminal pro B-type natriuretic peptide; WBC–white blood cells count. Bold: statistical significance <0.05.

**Table 3:** Echocardiographic parameters of the total cohort and data stratified by the history of myocardial infarction.

Variable	Total Cohort N = 90	ADHF without Prior MI N = 50	ADHF with Prior MI N = 40	*p*-Value *
LVEF, biplane Simpson, %	43.4 ± 16.4	46.3 ± 15.8	39.9 ± 16.6	0.066
LV mass, g	254.4 ± 95.7	250.2 ± 93.9	258.5 ± 97.5	0.686
LVMI, g/m^2^	119.0 ± 47.3	117.3 ± 46.5	120.7 ± 48.1	0.744
LV EDd, mm	57.9 ± 9.4	56.9 ± 8.5	58.9 ± 10.4	0.322
LV ESd, mm	42.6 ± 12.1	40.9 ± 11.3	44.6 ± 12.9	0.152
IVSd, mm	11 (10–13)	11 (10–13.5)	11 (10–12)	0.405
LV PWd, mm	10.9 ± 2.0	11.0 ± 1.9	10.9 ± 2.1	0.734
FS, %	27.3 ± 11.5	28.4 ± 11.4	25.9 ± 11.6	0.326
LV EDV, mL/m^2^ **	85.2 ± 32.3	80.4 ± 26.4	91.1 ± 38.0	0.142
LV ESV, mL/m^2^ **	45.0 ± 29.7	40.0 ± 25.4	51.3 ± 33.6	0.096
LA diameter, mm	49.9 ± 8.9	49.5 ± 9.7	50.3 ± 7.7	0.685
Aortic root diameter, mm	33.8 ± 5.1	33.3 ± 5.3	34.5 ± 4.9	0.295
LA/Ao ratio	1.49 ± 0.28	1.49 ± 0.31	1.48 ± 0.24	0.757

Values are mean ± SD or median (interquartile range); * Based on variable distribution, an independent samples *t*-test or the Mann–Whitney U test were used for group comparisons, as appropriate; ** Indexed to body surface area. Abbreviations: Ao—aortic root diameter; BSA—body surface area; EDd—end-diastolic diameter; EDV—end-diastolic volume; ESd—end-systolic diameter; ESV—end-systolic volume; FS—fractional shortening; IVSd—interventricular septum diameter; LA—left atrium; LV—left ventricle; LVEF—left ventricular ejection fraction; LVMI—left ventricular mass index; PWd—posterior wall diameter.

**Table 4:** Univariate beta estimates and results from multivariable linear regression showing associations of serum CST levels (ng/mL) with the clinical and laboratory parameters of interest.

Variable	Univariate β	*p*-Value *	B	SE	β	t	*p*-Value **
NYHA class	0.533	<0.001	0.361	0.082	0.491	4.257	**<0.001**
WHR	−0.335	0.012	−1.360	0.615	−0.237	−2.231	**0.026**
Heart rate at admission, bpm	−0.164	0.125	−0.933	0.418	−0.201	−1.960	**0.036**
Heart rate at rest, bpm	−0.189	0.098	−0.951	0.451	−0.242	−2.177	**0.030**
Total cholesterol, mmol/L	−0.108	0.268					
Triglycerides, mmol/L	−0.201	0.101					
HDL-c, mmol/L	−0.082	0.508					
LDL-c, mmol/L	−0.272	0.019	−0.089	0.040	−0.231	−2.123	**0.029**
Non-HDL cholesterol, mmol/L	−0.281	0.014	−0.094	0.045	−0.237	−2.298	**0.026**
NT-proBNP, pg/mL	−0.193	0.074					
hs-cTnI, ng/L	−0.260	0.015	−0.080	0.111	−0.221	−0.799	**0.030**
CRP, mg/L	0.147	0.490					
D-dimer, mg/L	−0.094	0.770					
Glucose, fasting, mmol/L	−0.165	0.159					
HbA1c, %	−0.264	0.023	−0.085	0.039	−0.235	−1.248	**0.027**

* Statistical significance for univariate beta estimate; ** Multivariable linear regression model when testing each variable was separately adjusted for age, body mass index, estimated glomerular filtration rate, systolic blood pressure, left ventricular ejection fraction, and weighted by sex and the history of myocardial infarction. Abbreviations: CRP-C—reactive protein; HDL—high-density lipoprotein; HbA1c—glycated hemoglobin; hs-cTnI—high sensitivity cardiac troponin I; LDL—low-density lipoprotein; NT-proBNP—N-terminal pro B-type natriuretic peptide; NYHA—New York Heart Association; WHR—Waist-to-Hip Ratio. Bold: statistical significance <0.05.

**Table 5:** Univariate beta estimates and results from multivariable linear regression showing associations of serum CST levels (ng/mL), with echocardiographic parameters.

Variable	Univariate β	*p*-Value *	B	SE	β	t	*p*-Value **
LVEF, %	0.323	0.010	0.700	0.299	0.271	2.350	**0.022**
LV mass, g	−0.312	0.019	−0.001	0.001	−0.249	−2.185	**0.031**
LVMI, g/m^2^	−0.301	0.022	−0.002	0.001	−0.237	−2.488	**0.015**
LV EDd, mm	−0.463	<0.001	−0.020	0.005	−0.341	−3.203	**0.001**
LV ESd, mm	−0.411	<0.001	−0.013	0.004	−0.311	−2.762	**0.005**
IVSd, mm	0.181	0.139					
LV PWd, mm	−0.165	0.180					
LV EDV, indexed to BSA, mL/m^2^	−0.375	<0.001	−0.002	0.001	−0.324	−3.211	**0.002**
LV ESV, indexed to BSA, mL/m^2^	−0.349	<0.001	−0.003	0.001	−0.328	−3.157	**0.002**
LA diameter, mm	−0.297	0.010	−0.012	0.005	−0.262	−2.415	**0.019**
Aortic root diameter, mm	−0.070	0.574					
LA/Ao ratio	−0.032	0.795					
LAEDV, mL	−0.171	0.251					
LAVI, mL/m^2^	−0.233	0.101					
Fractional shortening, %	0.292	0.021	0.011	0.003	0.255	2.198	**0.029**

* Statistical significance for univariate beta estimate; ** Multivariable linear regression model when testing each variable separately was adjusted for age, body mass index, estimated glomerular filtration rate, systolic blood pressure, and weighted by sex and the history of myocardial infarction; Abbreviations: Ao—aortic root diameter; BSA—body surface area; EDd—end-diastolic diameter; EDV—end-diastolic volume; ESd—end-systolic diameter; ESV—end-systolic volume; IVSd—interventricular septum diameter; LA—left atrium; LV—left ventricle; LVEF—left ventricular ejection fraction; LVMI—left ventricular mass index; PWd—posterior wall diameter. Bold: statistical significance <0.05.

## References

[1] Kurmani S., Squire I. (2017). Acute heart failure: Definition, classification and epidemiology. Curr. Heart Fail. Rep..

[2] Roger V.L. (2013). Epidemiology of heart failure. Circ. Res..

[3] Savarese G., Lund L.H. (2017). Global public health burden of heart failure. Card. Fail. Rev..

[4] Ibrahim N.E., Januzzi J.L. (2018). Established and emerging roles of biomarkers in heart failure. Circ. Res..

[5] Viquerat C.E., Daly P., Swedberg K., Evers C., Curran D., Parmley W.W., Chatterjee K. (1985). Endogenous catecholamine levels in chronic heart failure. Relation to the severity of hemodynamic abnormalities. Am. J. Med..

[6] Deng M.C., Brisse B., Erren M., Khurana C., Breithardt G., Scheld H.H. (1997). Ischemic versus idiopathic cardiomyopathy: Differing neurohumoral profiles despite comparable peak oxygen uptake. Int. J. Cardiol..

[7] Notarius C.F., Spaak J., Morris B.L., Floras J.S. (2007). Comparison of muscle sympathetic activity in ischemic and nonischemic heart failure. J. Card. Fail..

[8] Fung M.M., Salem R.M., Mehtani P., Thomas B., Lu C.F., Perez B., Rao F., Stridsberg M., Ziegler M.G., Mahata S.K. (2010). Direct vasoactive effects of the chromogranin A (CHGA) peptide catestatin in humans in vivo. Clin. Exp. Hypertens..

[9] Mahata S.K., O’Connor D.T., Mahata M., Yoo S.H., Taupenot L., Wu H., Gill B.M., Parmer R.J. (1997). Novel autocrine feedback control of catecholamine release. A discrete chromogranin a fragment is a noncompetitive nicotinic cholinergic antagonist. J. Clin. Invest..

[10] Mahata S.K., Kiranmayi M., Mahapatra N.R. (2018). Catestatin: A Master regulator of cardiovascular functions. Curr. Med. Chem..

[11] Borovac J.A., Dogas Z., Supe-Domic D., Galic T., Bozic J. (2019). Catestatin serum levels are increased in male patients with obstructive sleep apnea. Sleep Breath.

[12] Gaede A.H., Pilowsky P.M. (2012). Catestatin, a chromogranin A-derived peptide, is sympathoinhibitory and attenuates sympathetic barosensitivity and the chemoreflex in rat CVLM. Am. J. Physiol. Regul. Integr. Comp. Physiol..

[13] Wang X., Xu S., Liang Y., Zhu D., Mi L., Wang G., Gao W. (2011). Dramatic changes in catestatin are associated with hemodynamics in acute myocardial infarction. Biomarkers.

[14] Peng F., Chu S., Ding W., Liu L., Zhao J., Cui X., Li R., Wang J. (2016). The predictive value of plasma catestatin for all-cause and cardiac deaths in chronic heart failure patients. Peptides.

[15] Ottesen A.H., Carlson C.R., Louch W.E., Dahl M.B., Sandbu R.A., Johansen R.F., Jarstadmarken H., Bjoras M., Hoiseth A.D., Brynildsen J. (2017). Glycosylated chromogranin A in heart failure: Implications for processing and cardiomyocyte calcium homeostasis. Circ. Heart Fail..

[16] Meng L., Wang J., Ding W.H., Han P., Yang Y., Qi L.T., Zhang B.W. (2013). Plasma catestatin level in patients with acute myocardial infarction and its correlation with ventricular remodelling. Postgrad. Med. J..

[17] Liu L., Ding W., Li R., Ye X., Zhao J., Jiang J., Meng L., Wang J., Chu S., Han X. (2013). Plasma levels and diagnostic value of catestatin in patients with heart failure. Peptides.

[18] Zhu D., Wang F., Yu H., Mi L., Gao W. (2011). Catestatin is useful in detecting patients with stage B heart failure. Biomarkers.

[19] McKee P.A., Castelli W.P., McNamara P.M., Kannel W.B. (1971). The natural history of congestive heart failure: The Framingham study. N. Engl. J. Med..

[20] Ponikowski P., Voors A.A., Anker S.D., Bueno H., Cleland J.G., Coats A.J., Falk V., Gonzalez-Juanatey J.R., Harjola V.P., Jankowska E.A. (2016). 2016 ESC Guidelines for the diagnosis and treatment of acute and chronic heart failure: The task force for the diagnosis and treatment of acute and chronic heart failure of the European society of cardiology (ESC). Developed with the special contribution of the heart failure association (HFA) of the ESC. Eur. J. Heart Fail..

[21] Januzzi J.L., van Kimmenade R., Lainchbury J., Bayes-Genis A., Ordonez-Llanos J., Santalo-Bel M., Pinto Y.M., Richards M. (2006). NT-proBNP testing for diagnosis and short-term prognosis in acute destabilized heart failure: An international pooled analysis of 1256 patients: The international collaborative of NT-proBNP study. Eur. Heart J..

[22] Januzzi J.L., Camargo C.A., Anwaruddin S., Baggish A.L., Chen A.A., Krauser D.G., Tung R., Cameron R., Nagurney J.T., Chae C.U. (2005). The N-terminal Pro-BNP investigation of dyspnea in the emergency department (PRIDE) study. Am. J. Cardiol..

[23] Januzzi J.L., Chen-Tournoux A.A., Christenson R.H., Doros G., Hollander J.E., Levy P.D., Nagurney J.T., Nowak R.M., Pang P.S., Patel D. (2018). N-terminal pro–B-type natriuretic peptide in the emergency department: The ICON-RELOADED study. J. Am. Coll. Cardiol..

[24] Mosteller R.D. (1987). Simplified calculation of body-surface area. N. Engl. J. Med..

[25] Williams B., Mancia G., Spiering W., Agabiti Rosei E., Azizi M., Burnier M., Clement D.L., Coca A., de Simone G., Dominiczak A. (2018). 2018 ESC/ESH Guidelines for the management of arterial hypertension. Eur. Heart J..

[26] Levey A.S., Stevens L.A., Schmid C.H., Zhang Y.L., Castro A.F., Feldman H.I., Kusek J.W., Eggers P., Van Lente F., Greene T. (2009). A new equation to estimate glomerular filtration rate. Ann. Intern. Med..

[27] American Diabetes Association (2015). (2) Classification and diagnosis of diabetes. Diabetes Care.

[28] Surawicz B., Childers R., Deal Barbara J., Gettes Leonard S. (2009). AHA/ACCF/HRS Recommendations for the Standardization and interpretation of the electrocardiogram. Circulation.

[29] Lang R.M., Badano L.P., Mor-Avi V., Afilalo J., Armstrong A., Ernande L., Flachskampf F.A., Foster E., Goldstein S.A., Kuznetsova T. (2015). Recommendations for cardiac chamber quantification by echocardiography in adults: An update from the American Society of Echocardiography and the European Association of Cardiovascular Imaging. Eur. Heart J. Cardiovasc. Imaging.

[30] Folland E.D., Parisi A.F., Moynihan P.F., Jones D.R., Feldman C.L., Tow D.E. (1979). Assessment of left ventricular ejection fraction and volumes by real-time, two-dimensional echocardiography. A comparison of cineangiographic and radionuclide techniques. Circulation.

[31] Remme W.J. (1998). The sympathetic nervous system and ischaemic heart disease. Eur. Heart J..

[32] Liu L., Ding W., Zhao F., Shi L., Pang Y., Tang C. (2013). Plasma levels and potential roles of catestatin in patients with coronary heart disease. Scand. Cardiovasc. J..

[33] Taupenot L., Harper K.L., O’Connor D.T. (2003). The chromogranin-secretogranin family. N. Engl. J. Med..

[34] Helle K.B. (2004). The granin family of uniquely acidic proteins of the diffuse neuroendocrine system: Comparative and functional aspects. Biol. Rev. Camb. Philos. Soc..

[35] Gayen J.R., Gu Y., O’Connor D.T., Mahata S.K. (2009). Global disturbances in autonomic function yield cardiovascular instability and hypertension in the chromogranin a null mouse. Endocrinology.

[36] Pascual-Figal D.A., Manzano-Fernandez S., Boronat M., Casas T., Garrido I.P., Bonaque J.C., Pastor-Perez F., Valdes M., Januzzi J.L. (2011). Soluble ST2, high-sensitivity troponin T-and N-terminal pro-B-type natriuretic peptide: Complementary role for risk stratification in acutely decompensated heart failure. Eur. J. Heart Fail..

[37] Aimo A., Januzzi J.L., Vergaro G., Ripoli A., Latini R., Masson S., Magnoli M., Anand I.S., Cohn J.N., Tavazzi L. (2019). High-sensitivity troponin T, NT-proBNP and glomerular filtration rate: A multimarker strategy for risk stratification in chronic heart failure. Int. J. Cardiol..

[38] Ahmed A. (2007). A propensity matched study of New York Heart Association class and natural history end points in heart failure. Am. J. Cardiol..

[39] Ahmed A., Aronow W.S., Fleg J.L. (2006). Higher New York Heart Association classes and increased mortality and hospitalization in patients with heart failure and preserved left ventricular function. Am. Heart J..

[40] Muntwyler J., Abetel G., Gruner C., Follath F. (2002). One-year mortality among unselected outpatients with heart failure. Eur. Heart J..

[41] Wang D., Liu T., Shi S., Li R., Shan Y., Huang Y., Hu D., Huang C. (2016). Chronic administration of catestatin improves autonomic function and exerts cardioprotective effects in myocardial infarction rats. J. Cardiovasc. Pharmacol. Ther..

[42] Zhu D., Xie H., Wang X., Liang Y., Yu H., Gao W. (2015). Correlation of plasma catestatin level and the prognosis of patients with acute myocardial infarction. PLoS ONE.

[43] Meng L., Ye X.J., Ding W.H., Yang Y., Di B.B., Liu L., Huo Y. (2011). Plasma catecholamine release-inhibitory peptide catestatin in patients with essential hypertension. J. Cardiovasc. Med..

[44] Mahapatra N.R., O’Connor D.T., Vaingankar S.M., Hikim A.P., Mahata M., Ray S., Staite E., Wu H., Gu Y., Dalton N. (2005). Hypertension from targeted ablation of chromogranin A can be rescued by the human ortholog. J. Clin. Invest..

[45] Ying W., Mahata S., Bandyopadhyay G.K., Zhou Z., Wollam J., Vu J., Mayoral R., Chi N.W., Webster N.J.G., Corti A. (2018). Catestatin inhibits obesity-induced macrophage infiltration and inflammation in the liver and suppresses hepatic glucose production, leading to improved insulin sensitivity. Diabetes.

[46] Gallo M.P., Femmino S., Antoniotti S., Querio G., Alloatti G., Levi R. (2018). Catestatin induces glucose uptake and GLUT4 trafficking in adult rat cardiomyocytes. Biomed. Res. Int..

[47] Simunovic M., Supe-Domic D., Karin Z., Degoricija M., Paradzik M., Bozic J., Unic I., Skrabic V. (2019). Serum catestatin concentrations are decreased in obese children and adolescents. Pediatr. Diabetes.

[48] Bandyopadhyay G.K., Vu C.U., Gentile S., Lee H., Biswas N., Chi N.W., O’Connor D.T., Mahata S.K. (2012). Catestatin (chromogranin A (352–372)) and novel effects on mobilization of fat from adipose tissue through regulation of adrenergic and leptin signaling. J. Biol. Chem..

[49] Bandyopadhyay G.K., Mahata S.K. (2017). Chromogranin A regulation of obesity and peripheral insulin sensitivity. Front. Endocrinol..

[50] Durakoglugil M.E., Ayaz T., Kocaman S.A., Kirbas A., Durakoglugil T., Erdogan T., Cetin M., Sahin O.Z., Cicek Y. (2015). The relationship of plasma catestatin concentrations with metabolic and vascular parameters in untreated hypertensive patients: Influence on high-density lipoprotein cholesterol. Anatol. J. Cardiol..

[51] Kojima M., Ozawa N., Mori Y., Takahashi Y., Watanabe-Kominato K., Shirai R., Watanabe R., Sato K., Matsuyama T.A., Ishibashi-Ueda H. (2018). Catestatin prevents macrophage-driven atherosclerosis but not arterial injury-induced neointimal hyperplasia. Thromb. Haemost..

[52] Chen Y., Wang X., Yang C., Su X., Yang W., Dai Y., Han H., Jiang J., Lu L., Wang H. (2019). Decreased circulating catestatin levels are associated with coronary artery disease: The emerging anti-inflammatory role. Atherosclerosis.

[53] Wettersten N., Maisel A. (2015). Role of cardiac troponin levels in acute heart failure. Card. Fail. Rev..

[54] Januzzi J.L., Filippatos G., Nieminen M., Gheorghiade M. (2012). Troponin elevation in patients with heart failure: On behalf of the third Universal definition of myocardial infarction global task force: Heart failure section. Eur. Heart J..

[55] Angelone T., Quintieri A.M., Brar B.K., Limchaiyawat P.T., Tota B., Mahata S.K., Cerra M.C. (2008). The antihypertensive chromogranin a peptide catestatin acts as a novel endocrine/paracrine modulator of cardiac inotropism and lusitropism. Endocrinology.

[56] Imbrogno S., Garofalo F., Cerra M.C., Mahata S.K., Tota B. (2010). The catecholamine release-inhibitory peptide catestatin (chromogranin A344–363) modulates myocardial function in fish. J. Exp. Biol..

[57] Mazza R., Gattuso A., Mannarino C., Brar B.K., Barbieri S.F., Tota B., Mahata S.K. (2008). Catestatin (chromogranin A344–364) is a novel cardiosuppressive agent: Inhibition of isoproterenol and endothelin signaling in the frog heart. Am. J. Physiol. Heart Circ. Physiol..

[58] Dev N.B., Gayen J.R., O’Connor D.T., Mahata S.K. (2010). Chromogranin a and the autonomic system: Decomposition of heart rate variability and rescue by its catestatin fragment. Endocrinology.

